# Effects of Micronized Progesterone in Menopausal Hormone Replacement Therapy on Sleep

**DOI:** 10.1111/jog.70401

**Published:** 2026-07-15

**Authors:** Asuka Hirose, Masakazu Terauchi, Momo Hirata, Kazuko Matsuoka, Kenji Umayahara, Masaru Sakamoto, Naoyuki Miyasaka

**Affiliations:** ^1^ Department of Obstetrics and Gynecology Institute of Science Tokyo Hospital Tokyo Japan; ^2^ Department of Gynecology Kyoundo Hospital Tokyo Japan

**Keywords:** allopregnanolone, GABA, menopausal hormone therapy, sleep disorder, synthetic progestins

## Abstract

**Aim:**

In Japan, only synthetic progestins were available for menopausal hormone therapy until 2021. Since then, micronized progesterone has become available. Micronized progesterone binds to gamma‐aminobutyric acid type A receptors and exerts sedative and hypnotic effects. This study evaluated the effects of micronized progesterone on sleep in women receiving estrogen‐progestogen therapy.

**Methods:**

This retrospective study was approved by the institutional review board. Eligible participants were women who switched from synthetic progestins to micronized progesterone between December 2022 and September 2024. Women using sleep medications were excluded. Sleep quality was assessed using the Athens Insomnia Scale before switching, 3 months, and 1 year after initiating micronized progesterone. Progesterone was administered cyclically or continuously. In cyclic users, the Insomnia Severity Index was used to compare sleep during treatment and treatment‐free phases.

**Results:**

A total of 36 participants were included, of whom 22 completed a 1‐year follow‐up. At 3 months, sleep induction (*p* = 0.002) and awakenings during the night (*p* = 0.028) on the Athens Insomnia Scale improved, although the total score remained unchanged. At 1 year, no significant changes were observed. Among 11 participants with baseline scores ≤ 3, the total Athens Insomnia Scale scores significantly worsened at 3 months and 1 year. No significant differences in Insomnia Severity Index scores were observed between treatment and non‐treatment periods.

**Conclusions:**

Micronized progesterone improved sleep induction and reduced awakenings during the night at 3 months; however, benefits diminished by 1 year. Sleep conditions worsened in women without preexisting sleep disorders. Regular monitoring is recommended during long‐term micronized progesterone therapy.

## 1 Introduction

In Japan, only synthetic progestins were available for menopausal hormone therapy (MHT) until 2021. Medroxyprogesterone acetate (MPA) and dydrogesterone have commonly been prescribed. Since then, micronized progesterone (mP4) has become available for prescription. According to the medical package insert, mP4 should be taken before bedtime, as it may cause side effects such as drowsiness and dizziness. This is thought to result from the action of allopregnanolone (ALLO), a metabolite of progesterone that binds to gamma‐aminobutyric acid type A (GABA_A_) receptors and exerts sedative and hypnotic effects [1, 2]. Previous studies have shown that progesterone metabolites improve sleep parameters, and a systematic review and meta‐analysis of randomized controlled trials demonstrated that mP4 improves several sleep outcomes [3]. Progesterone metabolites have also been reported to induce sleep architecture changes similar to those observed with benzodiazepine‐like agents [4].

The GABA_A_ receptor functions as an inhibitory neurotransmitter receptor mediating anxiolytic, antidepressant, anesthetic, anticonvulsant, and analgesic effects. Progesterone metabolites bind to GABA_A_ receptors and enhance the effect of GABA, resulting in anxiolytic and sedative effects. Among these metabolites, ALLO is the best‐studied neuroactive steroid modulating neurotransmission [5]. ALLO has been implicated in postpartum depression, premenstrual syndrome, and premenstrual dysphoric disorder and is clinically used as brexanolone for the treatment of postpartum depression in the United States.

Progestogens prescribed for MHT include mP4 and synthetic progestins. mP4 is chemically identical to endogenous progesterone, whereas progestins are synthetic derivatives [6]. Oral progesterone, even micronized, exhibits wide interindividual variation in absorption and bioavailability. In contrast, synthetic progestins are rapidly absorbed, reach peak serum concentrations within 2–5 h, have longer half‐lives than progesterone, and maintain stable plasma levels during long‐term use. MPA, a derivative of 17α‐hydroxyprogesterone, acts not only at the progesterone receptor but also at androgen and glucocorticoid receptors. Dydrogesterone is a retroprogesterone, a stereoisomer of progesterone; its bent retrostructure confers high progesterone receptor selectivity [6].

The effects of mP4, MPA, and dydrogesterone on sleep have been examined in previous studies; however, no study has investigated sleep conditions after switching from synthetic progestins to mP4 in women already receiving MHT. Therefore, this study aimed to evaluate the effects of mP4 on sleep in women receiving estrogen–progestogen therapy.

## 2 Methods

### 2.1 Design

This retrospective study was conducted with the approval of the Ethics Committee of Kyoundo Hospital. The study included patients who were already receiving MHT for the treatment of menopausal symptoms and switched from synthetic progestins to mP4 between December 2022 and September 2024. Patients who continued mP4 therapy for at least 3 months were included in the analysis. Patients using sleep medications were excluded to minimize potential confounding effects on sleep outcomes. During the observation period, estrogen therapy remained unchanged after switching to mP4. Patients who discontinued mP4 therapy or lacked sufficient follow‐up data were excluded from the final analysis. This study was conducted in accordance with the principles set by the Declaration of Helsinki and its amendments [7].

### 2.2 Measures

The following patient characteristics were examined as background factors: age at the time of switching to mP4, age at the initiation of MHT, indication for MHT, type of estrogen preparation used, type of progestin used before switching to mP4, method of mP4 administration (cyclic or continuous), height, weight, and body mass index (BMI). Sleep quality was assessed using the Athens Insomnia Scale (AIS), which evaluates insomnia symptoms over the preceding 4 weeks, at three time points: before switching to mP4 (pre‐mP4), 3 months after the switch (3 months post‐mP4), and 1 year after the switch (1 year post‐mP4). The AIS was developed as a brief and easy‐to‐administer questionnaire for determining the severity of insomnia, as defined according to the International Classification of Diseases, Tenth Revision [8]. Its utility in clinical practice, research, and reliability were previously confirmed [9].

There are two mP4 administration regimens: cyclic and continuous. For patients receiving the cyclic regimen, the Insomnia Severity Index (ISI) was additionally used. Cyclic therapy consisted of 2 weeks of mP4 administration followed by 2 weeks without medication. Because the ISI evaluates insomnia symptoms over the preceding 2 weeks, it was considered appropriate for assessing sleep status specifically during the medication period in patients receiving cyclic therapy. In contrast, patients receiving continuous mP4 administration were evaluated using the AIS alone. The ISI is a brief instrument that assesses insomnia according to the criteria from the Diagnostic and Statistical Manual of Mental Disorders, Fourth Edition and the International Classification of Sleep Disorders [10]. It is being increasingly used in clinical and research activities. Its reliability, validity, and sensitivity to treatment responses have been documented in the general population and patients presenting with primary insomnia and insomnia in cancer settings [11, 12, 13].

Menopausal symptoms were assessed using the Menopausal Symptom Scale (MSS), which has been used and validated in previous studies [14], in which patients rate the severity of 10 menopausal symptoms at every visit. The MSS evaluates vasomotor symptoms (hot flush, perspiration, and chilliness), somatic symptoms (irregular heartbeat, headache or dizziness, tiredness, and aching joints or muscles), and psychological symptoms (insomnia, irritability, and depressed mood) using a four‐point Likert scale, depending on how often each symptom affects their daily life: none (never, 0 point), mild (rarely, 1 point), moderate (sometimes, 2 points), or severe (very often, 3 points). The MSS scores were calculated as the total score for the aforementioned symptoms.

### 2.3 Statistical Analyses

We compared the AIS and MSS scores at pre‐mP4 with those at 3 months post‐mP4 and 1 year post‐mP4 using a Wilcoxon signed‐rank test. Next, for patients on the cyclic regimen, ISI scores were compared at 3 months post‐mP4 and 1 year post‐mP4 to evaluate the differences between the medication‐ and medication‐free periods using a Wilcoxon signed‐rank test. In addition, a subanalysis was performed on patients with pre‐mP4 AIS score ≤ 3, who were considered to have no sleep problems. AIS scores at pre‐mP4 were compared with those at 3 months and 1 year post‐mP4.

Statistical analyses were performed using R version 4.2.0 (R Foundation for Statistical Computing, Vienna, Austria). Statistical significance was set at *p* < 0.05.

## 3 Results

A total of 36 women were included in the study, and 22 of them were evaluated 1 year post‐mP4 in addition to 3 months post‐mP4. Table 1 presents the baseline characteristics of the participants. The mean age was 55.0 years, and the mean age at initiation of MHT was 50.9 years. Regarding the progestin used, MPA was administered to two and 12 patients in the cyclic and continuous regimens, respectively, and dydrogesterone was administered to 10 and 12 patients in the cyclic and continuous regimens, respectively.

**TABLE 1:** Participant characteristics (36 patients).

Age at the time of switching to mP4 (y, mean ± SD)	55.0 ± 5.7
Height (cm, mean ± SD)	159.5 ± 4.8
Weight (kg, mean ± SD)	55.4 ± 8.2
BMI (kg/m^2^, mean ± SD)	21.9 ± 3.4
Age at MHT initiation (y, mean ± SD)	50.9 ± 3.8
Type of estrogen preparation used (*n*)	
Transdermal 17‐beta estradiol patch 0.72 mg/Same as above 0.36 mg/	21/8/
Transdermal 17‐beta estradiol gel 1.08 mg/Oral 17‐beta estradiol 1.0 mg/	3/3/
Transvaginal estriol 1 mg	1
Type of progestin used before switching to mP4 (*n*);	
Cyclic MPA (5 mg/day)/Continuous MPA (2.5 mg/day)/	2/12/
Cyclic dydrogesterone (10 mg/day)/Continuous dydrogesterone (5 mg/day)	10/12
AIS pre‐mP4 (mean ± SD)	5.0 ± 2.9

Abbreviations: AIS; Athens Insomnia Scale; BMI; body mass index; MHT; menopausal hormone therapy; MPA; medroxyprogesterone acetate; SD; standard deviation.

When comparing AIS scores pre‐mP4 and 3 months post‐mP4, there was no significant difference in the total score (Table 2). However, significant improvements in sleep induction and awakenings during the night were observed. No significant differences were observed in the overall quality of sleep, physical and mental functioning, or sleepiness during the day. At 1 year post‐mP4, there were no significant differences in any of the AIS components compared to pre‐mP4, including sleep induction and awakenings during the night. In the MSS analysis, including the insomnia item, no significant changes were observed in any items between pre‐mP4 and 3 months post‐mP4 or between pre‐mP4 and 1 year post‐mP4 (Table 3).

**TABLE 2:** AIS comparison before and after mP4 (36 patients).

	Pre‐mP4 (*n* = 36)	3 months post‐mP4 (*n* = 36)	*p* Pre vs. 3 months post mP4	1 year post‐mP4 (*n* = 22)	*p* Pre vs. 1 year post mP4
AIS total	5.0 ± 2.9	3.9 ± 2.2	0.08	4.6 ± 2.2	0.20
Sleep induction	0.7 ± 0.7	0.3 ± 0.4	0.002**	0.5 ± 0.7	0.05
Awakenings during the night	0.6 ± 0.6	0.3 ± 0.5	0.03*	0.4 ± 0.6	0.10
Final awakening earlier than desired	0.5 ± 0.6	0.5 ± 0.6	0.80	0.6 ± 0.7	0.60
Total sleep duration	1.0 ± 0.6	0.9 ± 0.6	0.48	0.9 ± 0.6	0.41
Overall quality of sleep	0.8 ± 0.6	0.7 ± 0.6	0.40	0.7 ± 0.5	0.23
Sense of well‐being during the day	0.3 ± 0.5	0.2 ± 0.4	0.23	0.3 ± 0.5	0.30
Functioning (physical and mental) during the day	0.3 ± 0.5	0.2 ± 0.4	0.57	0.3 ± 0.6	1.00
Sleepiness during the day	0.8 ± 0.4	0.8 ± 0.6	1.00	1.0 ± 0.5	0.23

*Note:* * *p* < 0.05, **p < 0.01. Wilcoxon singed‐rank test.

Abbreviations: AIS; Athens Insomnia Scale, mp4; micronized progesterone.

**TABLE 3:** MSS comparison before and after mP4 (36 patients).

	Pre‐mP4 (*n* = 36)	3 months post‐mP4 (*n* = 36)	*p*— Pre vs. 3 months post mP4	1 year post‐mP4 (*n* = 22)	*p*— Pre vs. 1 year post mP4
MSS total	10.6 ± 4.8	10.1 ± 4.4	0.40	10.8 ± 4.3	0.25
Hot flush	0.5 ± 0.7	0.6 ± 0.7	0.53	0.5 ± 0.7	0.59
Perspiration	0.8 ± 0.8	0.9 ± 0.8	0.39	0.6 ± 0.7	0.59
Chilliness	1.4 ± 0.8	1.2 ± 0.9	0.19	1.4 ± 0.8	0.31
Irregular heartbeat	0.7 ± 0.9	0.8 ± 0.8	0.64	0.7 ± 0.7	1.00
Insomnia	1.2 ± 0.8	1.1 ± 0.8	0.51	1.3 ± 0.8	0.61
Irritability	0.8 ± 0.9	0.7 ± 0.8	0.15	0.8 ± 0.8	0.42
Depressed mood	0.9 ± 0.8	0.8 ± 0.7	0.07	1.0 ± 1.0	0.59
Headache/dizziness	0.9 ± 0.9	0.7 ± 0.8	0.09	0.7 ± 0.7	0.31
Tiredness	1.5 ± 0.9	1.6 ± 0.9	0.50	1.8 ± 0.9	0.05
Aching joints/muscles	1.9 ± 0.9	1.9 ± 0.8	0.70	2.0 ± 0.9	0.83

Abbreviations: MSS; Menopausal Symptom Scale, mp4; micronized progesterone.

Next, we compared ISI scores between the medication‐ and medication‐free periods in patients using the cyclic regimens (Table 4). There were no significant differences in the ISI scores between the medication‐ and medication‐free periods at either 3 months or 1 year post‐mP4.

**TABLE 4:** ISI in cyclic users (12 patients).

	Medication periods	Medication‐free periods	*p*
3 months post‐mP4 (*n* = 12)	6.4 ± 3.4	5.8 ± 2.8	0.50
1 year post‐mP4 (*n* = 8)	4.8 ± 2.3	4.4 ± 2.6	0.85

Abbreviations: ISI; Insomnia Severity Index, mp4; micronized progesterone.

We then conducted a subgroup analysis of 11 participants whose AIS scores were ≤ 3 at pre‐mP4 (Table 5). At both 3 months and 1 year post‐mP4, no statistically significant changes were observed in the subscales, but the total AIS score had significantly worsened.

**TABLE 5:** Subgroup analysis—patients with AIS score three or less at baseline (11 patients).

	Pre‐mP4 (*n* = 11)	3 months post‐mP4 (*n* = 11)	*p*— Pre vs. 3 months post mP4	1 year post‐mP4 (*n* = 3)	*p*— Pre vs. 1 year post mP4
AIS total	1.5 ± 0.9	3.1 ± 2.2	0.05*	3.1 ± 1.3	0.03*
Sleep induction	0.2 ± 0.4	0.2 ± 0.4	1.00	0.1 ± 0.4	1.00
Awakenings during the night	0.1 ± 0.3	0.0 ± 0.0	1.00	0.3 ± 0.5	0.77
Final awakening earlier than desired	0.0 ± 0.0	0.2 ± 0.4	0.35	0.6 ± 0.5	0.07
Total sleep duration	0.5 ± 0.5	0.8 ± 0.6	0.23	0.6 ± 0.5	0.77
Overall quality of sleep	0.1 ± 0.3	0.5 ± 0.7	0.17	0.4 ± 0.5	0.35
Sense of well‐being during the day	0.0 ± 0.0	0.2 ± 0.4	0.35	0.1 ± 0.4	1.00
Functioning (physical and mental) during the day	0.0 ± 0.0	0.3 ± 0.5	0.15	0.1 ± 0.4	1.00
Sleepiness during the day	0.6 ± 0.5	1.0 ± 0.8	0.07	0.9 ± 0.7	0.35

Abbreviations: AIS; Athens Insomnia Scale, mp4; micronized progesterone.

* *p* < 0.05, Wilcoxon singed‐rank test.

## 4 Discussion

Many studies have examined the effects of mP4, MPA, and dydrogesterone on sleep; however, only three have directly compared mP4 with synthetic progestins. The first, by Marco Gambacciani et al. [15], compared three groups: a calcium–vitamin control group, a group receiving 0.3 mg conjugated estrogens (CE) plus 2.5 mg MPA, and a group receiving 0.3 mg CE plus 100 mg mP4 administered at bedtime. Sleep improved in both treatment groups compared to controls; however, the CE plus mP4 group showed a greater benefit, suggesting that mP4 may be more effective than MPA [15]. The second, by Montplaisir et al. compared two groups: one receiving 0.625 mg CE with 5 mg MPA and the other receiving 0.625 mg CE plus 200 mg mP4 [16]. Only mP4 improved sleep efficiency and reduced wake time, again suggesting superiority. The third, by Ekachai Leeangkoonsathian et al. [17], compared two groups: one receiving 1 mg of estradiol valerate plus 10 mg of dydrogesterone with one receiving 1 mg of estradiol valerate plus 100 mg of mP4. Both groups showed improved sleep with no significant difference. In our study, patients already receiving MHT with synthetic progestins were switched to mP4, enabling the direct evaluation of differences in sedative and hypnotic effects between synthetic and natural progestogens. Unlike previous studies, in which MHT was initiated at trial onset, our design minimized confounding by ongoing estrogen therapy, allowing for the assessment of the specific contribution of progestogens.

We found that mP4 significantly improved sleep induction and reduced awakenings during the night after 3 months. This effect is thought to result from the metabolism of mP4 to progesterone derivatives, including ALLO, which act on the GABA_A_ receptor. However, the sedative and hypnotic effects of these synthetic progestins remain unclear. In ovariectomized rats, 2 weeks of oral MPA increased ALLO levels [18]. More recently, in vitro studies showed that MPA and its metabolites enhance signal transduction in specific GABA_A_ receptor subtypes [19]. These findings suggest that synthetic progestins may modulate ALLO or exert sedative effects via GABA_A_.

In the MSS analysis, no significant changes were observed in any items, including the insomnia item, before and after mP4 administration. We speculate that mP4 may affect only specific aspects of sleep‐related problems, which may explain why significant changes were detected in the AIS, a more detailed assessment of sleep symptoms. Furthermore, switching from synthetic progestins to mP4 did not appear to affect menopausal symptoms other than sleep.

However, by 1 year, improvements in sleep were no longer evident, and the symptoms had returned to baseline. This transient benefit may reflect tolerance or a benzodiazepine‐like dependence. Similar to ALLO, benzodiazepines act via the GABA_A_ receptors to produce anxiolytic, sedative, hypnotic, and anticonvulsant effects. Long‐term benzodiazepine use can induce tolerance and dependence through the dopaminergic reward pathways and GABA receptor downregulation, mechanisms that may also explain the diminished efficacy of mP4 over time [20].

Furthermore, among patients on a cyclic regimen, no significant differences in sleep quality were found between the medication and medication‐free periods. Because patients using hypnotics were excluded, the prevalence of baseline sleep disorders in this cohort was likely low. In this population, mP4 did not appear to induce changes in sleep substantial enough to produce a significant difference between the treatment and withdrawal periods.

Finally, in the subgroup of patients with good baseline sleep quality, the total AIS score significantly worsened at both 3 months and 1 year. Although none of the individual subscales showed significant differences, this may have been due to reduced statistical power resulting from the small sample size. In patients who originally had no problems with sleep induction or awakenings during the night, the effects of mP4 may have been perceived more as discomfort than as a benefit. These findings highlight the importance of regular sleep monitoring and assessment of daytime functioning in women receiving mP4 therapy.

This study has several limitations. This study has several limitations. First, its retrospective design and lack of blinding may have introduced bias, particularly because drowsiness is a known effect of mP4 and may have influenced subjective sleep assessments. Second, the sample size was small. Third, sleep outcomes were evaluated only using self‐reported questionnaires without objective assessments such as polysomnography or actigraphy. Therefore, larger prospective controlled studies incorporating objective sleep measures are needed to clarify the effects of mP4 on sleep.

In conclusion, mP4 administration was associated with improvements in sleep induction and awakenings during the night at 3 months in women receiving MHT; however, these effects were not sustained after 1 year. Furthermore, among patients without preexisting sleep disturbance, the AIS total score worsened significantly at both 3 months and 1 year. Given the retrospective design, small sample size, lack of a control group, and reliance on subjective outcome measures, these findings should be considered exploratory and hypothesis‐generating. Prospective controlled studies with larger sample sizes are required to confirm the long‐term effects of mP4 on sleep.

## Author Contributions

**Asuka Hirose:** writing – original draft, methodology, conceptualization, investigation, validation, formal analysis, project administration. **Naoyuki Miyasaka:** writing – review and editing. **Masaru Sakamoto:** writing – review and editing. **Masakazu Terauchi:** writing – review and editing. **Momo Hirata:** writing – review and editing. **Kenji Umayahara:** writing – review and editing. **Kazuko Matsuoka:** writing – review and editing.

## Disclosure

An earlier version of this article was presented at the Asia‐Pacific Menopause Federation (APMF) 10th Scientific Meeting, which was held in Kuala Lumpur, Malaysia, on 25–27 July 2025.

## Ethics Statement

This study was approved by the Ethics Committee of Kyoundo Hospital (approval number: ER2024‐03).

## Consent

Informed consent was obtained by providing study information publicly and offering participants the opportunity to decline participation through an opt‐out procedure.

## Conflicts of Interest

The authors declare no conflicts of interest. Dr. Masakazu Terauchi is an Editorial Board member of the JOGR Journal and coauthor of this article. To minimize bias, he was excluded from all editorial decision‐making related to the acceptance of this article for publication.

## Data Availability

The data supporting the findings of this study contain sensitive clinical information obtained from a relatively small cohort of participants receiving menopausal hormone therapy. Although the data were anonymized prior to analysis, there remains a potential risk of participant re‐identification through combinations of variables such as age, menopausal status, treatment regimen, and sleep‐related outcomes. In addition, the informed consent approved by the institutional ethics committee did not include permission for unrestricted public data sharing. Therefore, the datasets are not publicly available due to privacy and ethical restrictions. However, anonymized data may be made available from the corresponding author upon reasonable request for scientifically valid research purposes, subject to approval by the institutional ethics committee where required.
